# Morbidity following emergency and elective cholecystectomy: a retrospective comparative cohort study

**DOI:** 10.1007/s00464-022-09103-2

**Published:** 2022-02-24

**Authors:** James Lucocq, Ganesh Radhakishnan, John Scollay, Pradeep Patil

**Affiliations:** Department of General and Upper GI Surgery, Ninewells Hospital, Dundee, UK

**Keywords:** Laparoscopic cholecystectomy, Morbidity, Emergency, Elective, Subtotal, Complication

## Abstract

**Introduction:**

An emergency laparoscopic cholecystectomy (EMLC) is commonly performed for all biliary pathology, yet EMLC can be challenging due to acute inflammation. Understanding the risks of EMLC is necessary before patients can make an informed decision regarding operative management. The aim of the present study was to compare rates of operative and post-operative outcomes between EMLC and elective LC (ELLC) using a large contemporary cohort, to inform the consent process and influence surgical decision making.

**Methods:**

All patients who underwent EMLC and ELLC in one UK health board between January 2015 and December 2019 were considered for inclusion. Data were collected retrospectively from multiple regional databases using a deterministic records-linkage methodology. Patients were followed up for 100 days post-operatively for adverse outcomes and outcomes were compared between groups using both univariate and multivariate analysis adjusting for pre-operative factors.

**Results:**

A total of 2768 LCs were performed [age (range), 52(13–92); M:F, 1:2.7]. In both the univariate and multivariate analysis, EMLC was positively associated with subtotal cholecystectomy (RR 2.0; *p* < 0.001), post-operative complication (RR 2.8; *p* < 0.001), post-operative imaging (RR 2.0; *p* < 0.001), post-operative intervention (RR 2.3; *p* < 0.001), prolonged post-operative hospitalisation (RR 3.8; *p* < 0.001) and readmission (RR 2.2; *p* < 0.001). EMLC had higher rates of post-operative mortality in univariate analysis (RR 10.8; *p* = 0.01).

**Discussion:**

EMLC is positively associated with adverse outcomes versus ELLC. Of course this study does not focus on a specific biliary pathology; nevertheless, it illustrates the additional risk associated with EMLC. This should be clearly outlined during the consent process but should be balanced with the risk of further biliary attacks. Further studies are required to identify particular patient groups who benefit from elective surgery.

An emergency laparoscopic cholecystectomy (EMLC) is commonly performed for all biliary pathology. The updated 2018 Tokyo guidelines recommend EMLC for mild acute cholecystitis [1]. Similarly early laparoscopic cholecystectomy is justified for both gallstone pancreatitis and biliary colic to reduce the risk of further biliary attacks [2, 3]. Nevertheless, EMLC remains challenging due to acute inflammation and some surgeons are reluctant to perform the operation [4].

Patients should have a complete understanding of the operative and post-operative morbidity following EMLC as part of an informed consent process. Although EMLC is a common operation, the listed risks and incidences on consent forms vary and provide inadequate detail [5–9]. Proceeding with EMLC without informed consent, exposes surgeons to litigation risk. The consent process should include rates of complications, conversion to open, subtotal cholecystectomy, use of drains, prolonged post-operative hospitalisation and re-admission.

As part of the consent process, patients should weigh up the benefits and risks of EMLC versus waiting until an elective LC (ELLC). Unfortunately, the majority of studies comparing outcomes between EMLC and ELLC have small sample sizes and it is likely that statistical differences in some peri-operative outcomes have been missed [10–13]. As a result the implications of EMLC remain uncertain and require further investigation.

The aim of the present study was to compare rates of adverse operative and post-operative outcomes between EMLC and ELLC using a large contemporary cohort, to inform the consent process and influence surgical decision making.

## Materials and methods

### Population cohort

**Fig. 1:**
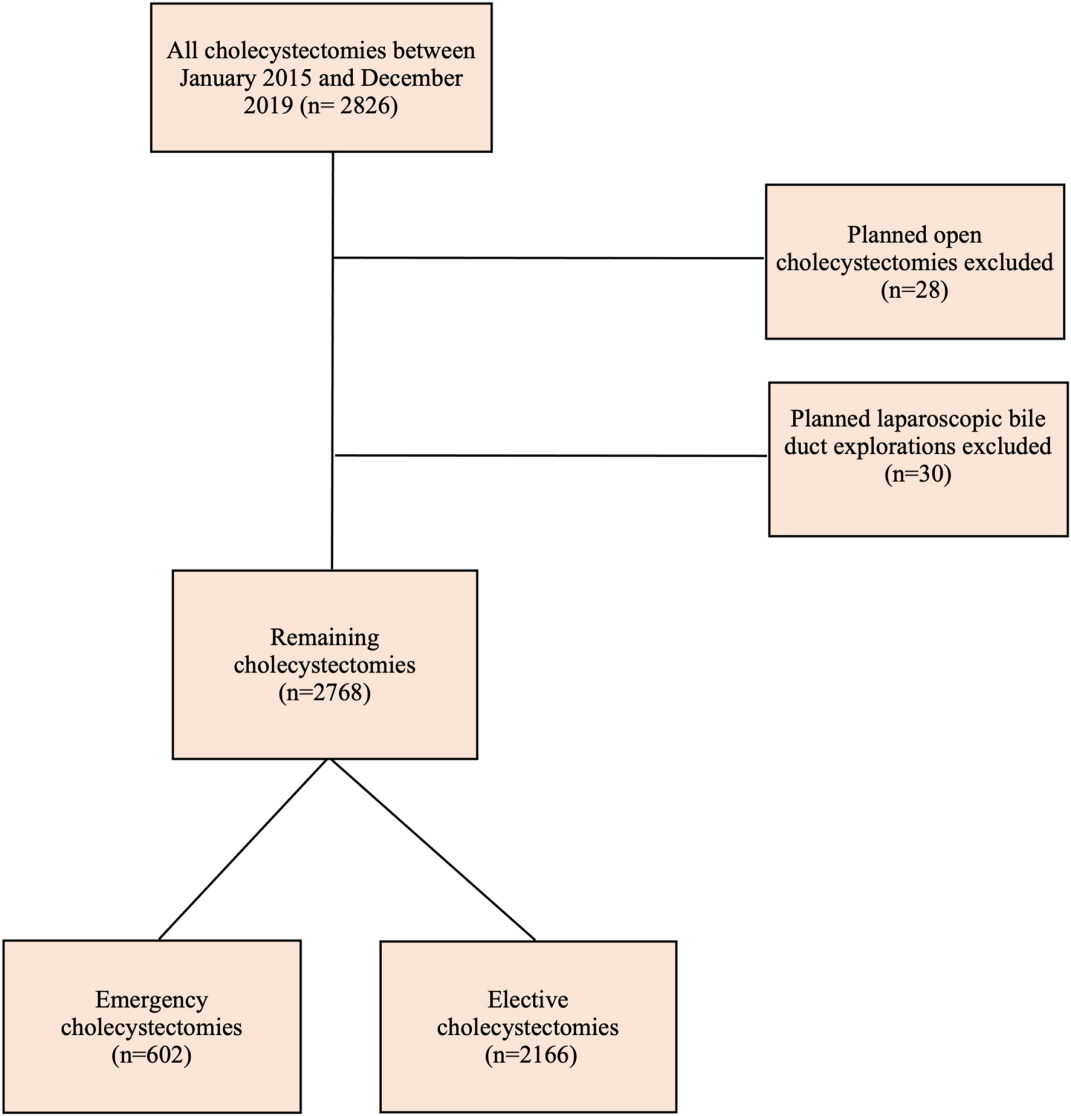
Study design

EMLC and ELLC performed for biliary pathology across three surgical units between January 2015 and December 2019, performed by 25 general surgical consultants were included in the study. The surgical units were located in a defined geographical region with a stable population of more than 490,000 people with less than a 1% migration rate [14]. Planned open cholecystectomies and bile duct explorations were excluded from analysis; laparoscopic cholecystectomies converted to open cholecystectomies and unplanned bile duct explorations were retained (Fig. 1). Indications for both EMLC and ELLC included all symptomatic biliary pathology (e.g. biliary colic, cholecystitis, gallstone pancreatitis). Ethical approval was granted by the regional information governance committee. Patient written consent was not required.

### Data

Data were collected retrospectively from multiple regional databases using a deterministic records-linkage methodology. Patients were tracked between databases using a unique patient identifier. Databases included ‘Clinical Portal’, ‘Integrated Clinical Environment’, ‘Picture Archiving and Communication System’ and ‘Referral Management System’ for data retrieval.

Pre-operative data included demographics, American Society of Anaesthesiology score (ASA), number of admissions, indication, pre-operative ERCP and pre-operative cholecystectomy. Operative data included intra-operative complications, use of drains, subtotal cholecystectomy and conversion to open. Post-operative data included complications (Clavien–Dindo ≥ 2), further imaging (e.g. MRCP, CT A/P), interventions (e.g. ERCP, return to theatre), post-operative length of stay and readmissions. All patients were followed up for 100 days post-operatively. A prolonged post-operative hospitalisation was defined as an in-patient stay of at least 3 days following cholecystectomy.

### Analysis

The rates of outcomes were compared between EMLC and ELLC groups using univariate analysis and multivariate analysis. Chi-squared, Fisher-exact and Mann–whitney *U* tests were conducted. Multivariate logistic regression models were created to find the association between EMLC and operative/post-operative adverse outcomes. These models adjusted for the following pre-operative variables: sex, age (< 40; 40–60; > 60), number of admissions (0; 1; 2; ≥ 3), ASA (1; 2; ≥ 3), cholecystitis, gallstone pancreatitis, choledocholithiasis, pre-operative ERCP and pre-operative cholecystostomy. All statistical tests were carried out using the STATA/IC 2019 statistical package.

Within the EMLC group, the median time from presentation to laparoscopic cholecystectomy was calculated. Adverse outcomes were compared between the early group (< 48 h) and the late group (> 48 h).

## Results

2768 cholecystectomies were included in the study (median age, 53 years; range, 13–92; Male:Female, 1:2.7). EMLC and ELLC were performed in 602 and 2166 cases, respectively. Comparison of pre-operative and operative data between EMLC and ELLC are displayed in Table 1 and 2, respectively. Patients undergoing EMLC compared to ELLC were younger (*p* < 0.001), underwent a pre-operative MRCP more frequently (*p* < 0.001), and were less likely to have a pre-operative abdominal ultrasound (USS) (*p* = 0.004). EMLC patients were more likely to have either cholecystitis or gallstone pancreatitis versus biliary colic (*p* < 0.001) and had less previous admissions (*p* < 0.001) (Table 1).

**Table 1:** Comparison of pre-operative data between EMLC and ELLC; univariate analysis

	ELLC	EMLC	*p*-value
Median age (range), years	54 (13–92)	50 (15–92)	< 0.001
Male:Female	1:2.7	1:2.6	0.93
ASA, (%)			
1	726 (33.5)	204 (33.9)	0.87
2	1229 (56.7)	344 (57.1)	0.86
≥ 3	211 (9.7)	53 (8.8)	0.49
Indication, (%)			
Biliary colic	1295 (59.8)	218 (36.2)	< 0.001
Cholecystitis	703 (32.5)	272 (45.2)	< 0.001
Gallstone pancreatitis	91 (4.2)	90 (15.0)	< 0.001
Other (including choledocholithiasis, biliary dyskinesia)	77 (3.6)	22 (3.7)	0.91
Imaging, (%)			
USS abdomen	2087 (96.4)	564 (93.7)	0.004
MRCP	651 (30.1)	270 (44.9)	< 0.001
CT abdomen/pelvis	308 (14.2)	99 (16.4)	0.17
Pre-operative radiological findings, (%)			
Thickened gallbladder wall	668 (30.8)	261 (43.4)	< 0.001
Pericholecystic fluid	275 (12.7)	121 (20.1)	< 0.001
CBD stones	211 (9.7)	61 (10.1)	0.78
Pre-operative ERCP, (%)	221 (10.2)	58 (9.6)	0.68
Pre-operative cholecystostomy, (%)	36 (1.7)	4 (0.7)	0.07
Number of previous biliary-related admissions, (%)			
1	741 (34.2)	104 (17.3)	< 0.001
2	133 (6.1)	18 (3.0)	0.003
≥ 3	41 (1.9)	10 (1.7)	0.71
Previous failed cholecystectomy, (%)	19 (0.9)	2 (0.3)	0.17

**Table 2:** Comparison of operative data between EMLC and ELLC; univariate analysis

	ELLC	EMLC	Relative risk in EMLC	*p*-value
Median operative time, minutes (range)	72 (27–351)	82 (50–275)	–	< 0.001
LC converted to open (CTO), (%)	26 (1.2)	5 (0.8)	0.7	0.45
Subtotal cholecystectomy, (%)	60 (2.8)	34 (5.6)	2.0	< 0.001
Laparoscopic	54 (2.5)	34 (5.6)	2.3	< 0.001
CTO	6 (0.3)	0 (0.0)	–	–
Intra-operative cholangiogram, (%)	31 (1.4)	12 (2.0)	1.4	0.32
Detection of stone	7 (0.3)	5 (0.8)	2.6	0.09
Unplanned bile duct exploration	1 (0.0)	0 (0.0)	–	–
Placement of intra-operative drain, (%)	132 (6.1)	73 (12.1)	2.0	< 0.001
Intra-operative complications, (%)	42 (1.9)	15 (2.5)	1.3	0.40

### Operative outcomes

EMLC had longer operation time (82 min versus 72 min; *p* < 0.001), higher rates of subtotal cholecystectomy (RR 2.0; *p* < 0.001) and higher rates of intra-operative drains (RR 2.0; *p* < 0.001). There was no significant difference in rates of conversion to open or rates of intra-operative complication between EMLC and ELLC groups *(p* > 0.05) (Table 2). There were four bile duct injuries (0.18%) in the ELLC group and 0 bile duct injuries in the EMLC group (*p* = 0.58). Only one bile duct injury was a complete transection.

In the multivariate analysis, EMLC was positively associated with subtotal cholecystectomy (OR 2.01; *p* = 0.004), but no association was found with conversion to open or intra-operative complications (Table 5).

In the EMLC group, the median time from admission to operation was 3 days. There was no significant difference in rates of subtotal cholecystectomy or conversion to open between the early EMLC and late EMLC group (Table 3). The rate of intra-operative drain insertion was higher in the early EMLC group (RR = 1.6, *p* = 0.03).

**Table 3:** Comparison of peri-operative outcomes between early EMLC and late EMLC; univariate analysis

Adverse outcome (%)	Early EMLC (< 48 h), *n* = 290	Late EMLC (> 48 h), *n* = 312	Relative risk in early EMLC	*p*-value
LC converted to open (CTO)	2 (0.7)	3 (1.0)	0.7	0.72
Subtotal cholecystectomy	16 (5.5)	18 (5.8)	1.0	0.89
Placement of intra-operative drain	44 (15.2)	29 (9.3)	1.6	0.03
Intra-operative complications	4 (1.4)	11 (3.5)	0.4	0.09
Post-operative complications	38 (13.1)	46 (14.7)	0.9	0.56
Further imaging	46 (15.9)	50 (16.0)	1.0	0.96
Further intervention	19 (6.6)	18 (5.8)	1.1	0.69
Prolonged post-operative hospitalisation (≥ 3 days)	80 (27.6)	73 (23.4)	1.2	0.24
Readmission	37 (12.8)	42 (13.5)	0.9	0.80
Post-operative mortality	2 (0.7)	1 (0.3)	2.2	0.52

### Post-operative outcomes

Post-operative ERCP was required in 59 patients (2.1%), return to theatre in 30 patients (1.1%) and interventional radiological drainage in 10 patients (0.4%). Of those who returned to theatre, 18 patients underwent a laparoscopy and 9 patients a laparotomy.

EMLC patients were more likely to have post-operative complications (RR 2.8; *p* < 0.001), require further imaging (RR 2.0; *p* < 0.001), further intervention (RR 2.3; *p* < 0.001), prolonged post-operative hospitalisation (RR 3.8; *p*  < 0.001) and be re-admitted (RR 2.1; *p*  < 0.001) (Table 4). Rates of post-operative mortality were higher in the EMLC (RR 10.8; *p* = 0.01).

**Table 4:** Comparison of adverse post-operative outcomes between EMLC and ELLC; univariate analysis

Adverse post-operative outcome (%)	ELLC	EMLC	Relative risk in EMLC	*p*-value
Post-operative complications	107 (4.9)	84 (14.0)	2.8	< 0.001
Further imaging	175 (8.1)	96 (15.9)	2.0	< 0.001
Further intervention	59 (2.7)	37 (6.1)	2.3	< 0.001
Prolonged post-operative hospitalisation (≥ 3 days)	144 (6.6)	153 (25.4)	3.8	< 0.001
Readmission	132 (6.1)	79 (13.1)	2.2	< 0.001
Post-operative mortality	1 (0.05)	3 (0.5)	10.8	0.01

More specifically, the rates of post-operative collection in the EMLC and ELLC were 4.2% (25) and 2.0% (44), respectively (*p* < 0.001). Rates of post-operative bile leak were 3.8% (23) in the EMLC group and 1.6% (34) in the ELLC group (*p* < 0.001). Rates of pancreatitis were higher following EMLC (2.5% (15) versus 0.5% (10); *p* < 0.001).

In the multivariate analysis, EMLC patients were more likely to have post-operative complications (OR 1.56; *p* = 0.008), require further imaging (OR 1.96; *p* < 0.001), require further intervention (OR 2.44; *p* < 0.001), have a prolonged post-operative hospitalisation (OR 5.53; p < 0.001) and require readmission (OR 2.1; *p* < 0.001) (Table 5).

**Table 5:** Multivariate logistic regression, association between EMLC and operative/post-operative adverse outcomes

Adverse outcome	OR	Std. Err	*Z*	*p*-value	95% CI
Conversion to open					
EMLC	0.60	0.31	− 0.98	0.33	0.22–1.66
Subtotal cholecystectomy					
EMLC	2.01	0.49	2.89	0.004	1.25–3.24
Intra-operative complication					
EMLC	1.12	0.38	0.34	0.73	0.58–2.17
Post-operative complications					
EMLC	1.56	0.26	2.67	0.008	1.13–2.16
Post-operative imaging					
EMLC	1.96	0.28	4.64	< 0.001	1.47–2.61
Post-operative intervention					
EMLC	2.44	0.56	3.87	< 0.001	1.55–3.82
Prolonged post-operative hospitalisation					
EMLC	5.53	0.82	11.56	< 0.001	4.14–7.39
Readmission					
EMLC	2.1	0.34	4.64	< 0.001	1.53–2.87

Overall 36.7% of patients undergoing EMLC had a non-standard outcome, with either a subtotal cholecystectomy, conversion to open, intra-operative complication, post-operative complication, prolonged post-operative hospitalisation, post-operative imaging, post-operative intervention or readmission. There was no significant difference in post-operative outcomes between the early EMLC and late EMLC groups.

## Discussion

EMLC is positively associated with adverse outcomes when compared to ELLC, even once adjusting for pre-operative patient-specific variables. Rates of subtotal (5.6%), post-operative complication (14.0%), post-operative imaging (15.9%), intervention (6.1%), prolonged post-operative hospitalisation (25.4%) and readmission (13.1%) may be higher than anticipated. Furthermore, 36.7% of EMLC patients have one of the above unplanned adverse outcomes.

The cumulative risk represented by the above figures needs to be acknowledged and should be conveyed to the patient forming part of the informed consent process. The details explained during the consent of a laparoscopic cholecystectomy vary and are often incomplete [8, 9]. Incorporating the listed incidences into the consent process will improve patient awareness of the likely operative and post-operative outcomes and prepare patients for the possibility of a complicated post-operative course. This may influence a patients decision to proceed with EMLC, and they may instead prefer the option of a delayed procedure once the acute episode has settled. Highlighting the significant risks of EMLC may also help mitigate litiation risk following LC.

In the meta-analysis outlined in the 2018 Tokyo Guidelines, outcomes following early and delayed LC for acute cholecystitis have been compared. Key outcome measures included operating times, incidence of bile duct injury, length of hospital stay and overall cost of treatment. Although rates of bile duct injury are comparable between groups, there is not much emphasis on other forms of post-operative morbidity. Furthermore, the linked studies unfortunately have very small samples sizes and it is probable that any statistical difference in post-operative morbidity between the two groups would not be identified [15].

A number of large-cohort studies have arrived at similar conclusions to our study. Giger et al. reported outcomes of 22,953 patients found EMLC to be positively associated with post-operative local complication (*p* = 0.003) and post-operative systemic complications (*p* < 0.001) [16]. In comparison, our study was able to control for more pre-operative factors such as number of patient admissions, indication and pre-operative interventions. The CholeS study group performed in 2016 also found EMLC to be strongly associated with re-admission, post-operative complications and post-operative imaging and intervention [17]. Similar findings were recorded from the Swedish Registry (*n* = 63,685) who found significantly higher rates of intra-operative bleeding, post-operative complications and longer post-operative hospitalisation in the EMLC group [18]. The above large-cohort studies imply that a particular set of patients with biliary pathology will benefit from ELLC as compared to EMLC, at least with respect to post-operative complications. It is implied that operating during an acute episode with active inflammation will render the operation more challenging (confirmed with longer operation times and higher rates of subtotal) and will expose patients to an elevated risk of post-operative problems. Certainly our data indicate that post-operative imaging, intervention and conservative management with antibiotics are required more frequently following EMLC for reasons such as the higher incidence of intra-operative complication, post-operative complication, subtotal cholecystectomy and readmission.

The rate of subtotal cholecystectomy in the EMLC group was significant (5.6%), yet there were no bile duct injuries in this group. Although the rates of intra- and post-operative complications were higher in the EMLC group, this can be regarded as evidence that a subtotal cholecystectomy can be performed without significant risk of bile duct injury, even in the emergency setting.

Of the ELLC group, 1.7% of patients underwent a pre-operative cholecystostomy. Although the present study did not aim to determine the utility of cholecystectomy, the implications are that cholecystostomy can be used as a bridge to an elective cholecystectomy and avoid a high-risk EMLC. Further analysis is required to identify specific groups who benefit from cholecystostomy before proceeding to ELLC.

By definition all EMLC had an admission to hospital, whereas a lower proportion of the ELLC patients were admitted to hospital. One could argue that admitted patients had more severe pathology and therefore this patient group were more likely to have worse operative and post-operative outcomes. Although this is feasible, the multivariate logistic regression models have controlled for the number of patient admissions and the models determine the impact of specifically performing the cholecystectomy during the acute episode versus at a later date.

Of course this study does not deal specifically with a particular biliary pathology; yet, it illustrates the additional risk associated with emergency cholecystectomy. Further studies are required to highlight patient groups who specifically benefit from an ELLC in terms of a less complicated peri-operative course. Of course, at all times the reduced risk of ELLC should be balanced with the risk of further biliary attacks and further difficulty of performing LC on a patient with multiple episodes of inflammation. If ELLC is offered, it remains that surgeons should aim to achieve this in a timely manner following discharge to minimise risk of further admission.
